# A Novel Method in the Stratification of Post-Myocardial-Infarction Patients Based on Pathophysiology

**DOI:** 10.1371/journal.pone.0130158

**Published:** 2015-06-19

**Authors:** Ben He, Heng Ge, Fan Yang, Yujun Sun, Zheng Li, Meng Jiang, Yiting Fan, Jun Pu, Xuedong Shen

**Affiliations:** Department of Cardiology, Renji Hospital, School of Medicine, Shanghai Jiaotong University, Shanghai, China; Temple University, UNITED STATES

## Abstract

**Objectives:**

We proposed that the severity of ST-segment elevation myocardial infarction (STEMI) could be classified based on pathophysiological changes.

**Methods:**

First-STEMI patients were classified within hospitalization. Grade 0: no detectable myocardial necrosis; Grade 1: myocardial necrosis without functional and morphological abnormalities; Grade 2: myocardial necrosis with reduced LVEF; Grade 3: reduced LVEF on the basis of cardiac remodeling; Grade 4: mitral regurgitation additional to the Grade-3 criteria.

**Results:**

Of 180 patients, 1.7, 43.9, 26.1, 23.9 and 4.4% patients were classified as Grade 0 to 4, respectively. The classification is an independent predicator of 90-day MACEs (any death, resuscitated cardiac arrest, acute heart failure and stroke): the rate was 0, 5.1, 8.5, 48.8 and 75% from Grade 0 to 4 (p<0.001), respectively. The Grade-2 patients were more likely to have recovered left ventricular ejection fraction than the Grade-3/4 patients did after 90 days (48.9% vs. 19.1%, p<0.001). Avoiding complicated quantification, the classification served as a good reflection of infarction size as measured by cardiac magnetic resonance imaging (0±0, 15.68±8.48, 23.68±9.32, 36.12±11.35 and 40.66±14.33% of the left ventricular mass by Grade 0 to 4, P<0.001), and with a comparable prognostic value (AUC 0.819 vs. 0.813 for infarction size, p = 0.876 by C-statistics) for MACEs.

**Conclusions:**

The new classification represents an easy and objective method to scale the cardiac detriments for STEMI patients.

## Introduction

Thanks for the worldwide highlight and availability of therapeutic innovations for ST-segment Elevation Myocardial Infarction (STEMI), myocardial injury following STEMI tends to polarize[1,2]: the proportion of patients with minor cardiac detriments increases gradually, some even only experiencing transient EKG changes and slight elevation of biomarkers (abortive infarction), so that a considerable percentage of patients merely manifest with tissue injury rather than detectable functional or morphological cardiac abnormities. Although being referred to the same term of “myocardial infarction” according to the universal definition[3], the prognostic expectations of these patients are quite different from their counterparts who suffer from extensive infarctions. Regarding this, scaling the real impact of STEMI on individual patients is important.

Nevertheless, current evaluations are still discretely based on functional (i.e. left ventricular ejection fraction, LVEF) [4,5], symptomatic (i.e. NYHA classification) and clinical characteristics (i.e. age, sex, morbidities, et al)[6,7]. These factors may be arbitrary and changeable (like the symptom), or experiences-dependent (like the clinical characteristics), so that different conclusions may be made for the same patient. Moreover, some of the indicators even lose their values in minor infarctions; for example, LVEF cannot further differentiate patients with preserved contractions.

To achieve satisfactory reproducibility and validity, the ideal evaluation may follow the progressive pathophysiological changes after STEMI. Such a process is composed of several advancing stages that are predictable in each patient according to the severity of cardiac detriments[8–11]: i.e. myocardial edema caused by ischemia, permanent myocardial necrosis resulting from prolonged blood blockage, initial contracting dysfunction mostly due to myocardial stunning, compensatory cardiac remodeling triggered by extensive infarction, and the final cardiac decompensation. Meanwhile, the modern advancements of new cardiac assessing technologies allows more precise determination of these pathophysiologic stages. For example, the cardiac magnetic resonance (CMR) can visualize the tissue injury that is undetectable by traditional echocardiography[12]. Therefore, we believe that a classification based on pathophysiological stages may make up the insufficient evaluations for less-injured patients and represent a more subtle and comprehensive stratification of cardiac detriments. Considering the prognostic impact of pathophysiological progress, the method will not only facilitate information-interchange among doctors, self-awareness of the disease for patients and objective evaluation for post-STEMI labor capacity, but also improve the quality of cardiac rehabilitation.

**Fig 1:**
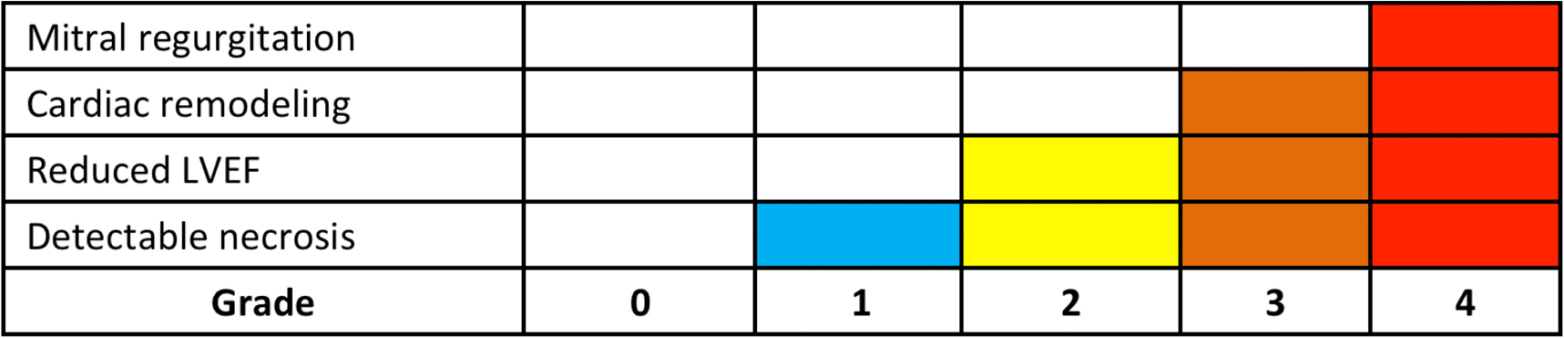
Post-STEMI patients is stratified based on graded pathophysiological criteria.

To validate the hypothesis, we stratified a cohort of STEMI patients into five groups: those without detectable myocardial necrosis, those with clear myocardial necrosis but are absent of functional and morphological abnormities, those with necrosis and demonstrating reduced LVEF, those with LVEF reduction on the basis of significant cardiac remodeling, and those with remarkable mitral regurgitation in addition to morphological and functional detriments (Fig 1). Our results have suggested that, by the proposed classification, the severity of STEMI can be clearly stratified, which is closely correlated with patient’s prognosis.

## Methods

### Study population

From May 2012 to March 2014, first-time-STEMI patients who received reperfusion therapy within 12h of symptom onset were included. The only exclusion criterion is a missing of CMR data within hospitalization.

STEMI was defined as a chest pain lasting ≧ 30 minutes together with an ST-segment elevation in ≧ 2 contiguous leads on a standard 12-lead electrocardiogram (≥2 mm in precordial leads and ≥1mm in the limb leads).

All patients received reperfusion therapy by either a primary PCI (PPCI) or a pharmacoinvasive strategy (initial thrombolysis plus PCI of residual stenosis after 3–24 hours). Standard therapeutic regimes were applied to every patient according to the 2013 ACCF/AHA Guideline for the management of STEMI[13].

### Definition of the classification

**Fig 2:**
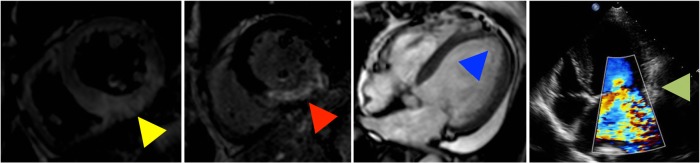
Examples for CMR findings of myocardial edema, necrosis, cardiac remodeling and echocardiography finding of severe mitral regurgitation (from left to right). The absence of detectable myocardial necrosis (red arrow head) is a key criterion for Grade-0 patients, for whom, myocardial edema (yellow arrow head) can be the only finding in the acute phase. Cardiac remodeling is defined as an expanded left ventricle (blue arrow head). Apparent mitral regurgitation (green arrow head) on the basis of cardiac remodeling is indicative of Grade 4.

Patients were classified by the following criteria: Grade 0: no detectable myocardial necrosis; Grade 1: presence of myocardial necrosis but without functional and morphological abnormalities; Grade 2: myocardial necrosis accompanied with reduced LVEF (LVEF<55%); Grade 3: with reduced LVEF on the basis of cardiac remodeling (≧15% increase of left ventricular end-diastolic volume (LVEDV) compared to normal limitation[14]); Grade 4: with medium or severe mitral regurgitation additional to the Grade-3 criteria. To achieve an equal definition and comparison, cardiac necrosis, LVEF and LVEDV were all assessed by CMR, while the degree of mitral regurgitation and follow-up cardiac improvements were determined by echocardiography. Imaging examples for grades are illustrated in Fig 2.

### CMR protocol and data analysis

CMR was performed at a median of 5.5 days after reperfusion. Images were acquired using an EKG-gated 3.0 Tesla scanner (Achieva, Philips Healthcare, The Netherlands).

The scanning protocol was described previously [15]. In brief, cine images were acquired using a balanced steady state free precession sequence (TR/TE 3.2/1.6ms, 30 phases, voxel size 2.0*1.6*8mm^3^). Myocardial edema was detected using a black blood T2 short tau inversion-recovery sequence (T2W-STIR, TR/TE 2 R-R intervals/75ms, voxel size 2.0*1.6*8 mm^3^). Myocardial necrosis was detected by late gadolinium enhancement (LGE) using a 3D inversion recovery segmented gradient echo sequence (TR/TE 3.5/1.7ms, temporal resolution 190ms, voxel size 1.5*1.7*10mm^3^ interpolated into 0.74*0.74*5mm^3^) 10 minutes after contrast injection (0.2mmol/kg, Magnevist, Bayer HealthCare Pharmaceuticals Inc., Germany). Individual optimized inversion time was carefully chosen in a Looklock sequence in order to null the signal of normal myocardium.

CMR data were inspected using commercial software (QMass MR 7.5, Medis Medical Imaging System, The Netherland). Left ventricular (LV) geometric and functional parameters were calculated on short-axis-view cine images. Myocardial edema and necrosis were determined as high-signal areas compared with remote non-infarcted myocardium on T2W-STIR and LGE images, respectively (≧2 SDs for edema and ≧5 SDs for necrosis). Infarction sizes were quantified and expressed as percentages of LV myocardial volume.

### Echocardiography protocol and data analysis

Echocardiography was performed on the same day of CMR and 90 days later (median 96.5 days, interquartile range 87.5 to 103.4 days), respectively, using a Vivid E9 scanner (GE Vingmed Ultrasound, Horten, Norway).

Images were analyzed using commercial software (Echopac, GE Vingmed Ultrasound, Horten, Norway). LVEF was calculated on short-axis-view images by the Simpson method; the degree of mitral regurgitation was categorized as mild, medium or severe according to the area ratio between regurgitation jet and left atrium [16]. A medium or severe regurgitation was defined as “apparent mitral regurgitation”.

### Events and Follow-up

Major cardiovascular adverse events (MACEs) were defined as a composite of any death, resuscitated cardiac arrest, acute heart failure (with typical manifestations of pulmonary edema), and stroke. In-hospital events were immediately recorded while the 90-day events were determined by telephone follow up with a pre-designed questionnaire.

### Ethics statement

The study protocol conforms to the ethical guidelines of the 1975 Declaration of Helsinki and was approved by the Human Research Ethic Committee of Renji Hospital, Shanghai Jiaotong University School of Medicine. All patients signed a written informed consent before inclusion.

### Statistical analysis

Continuous variables are expressed as mean ± standard deviation (SD). Categorical variables are expressed as the number and percentage of patients. Comparisons between two groups were performed by Student’s t-test in terms of continuous variables or by Chi-square/Fisher’s exact test for categorical variables. Continuous variables among several groups were compared by one-way ANOVA. Correlations between variables were assessed by Spearman’s correlation coefficients. To adjust the impact of baseline characteristics on prediction of MACEs, a logistic regression analysis with stepwise inclusion was performed including all possible influential factors. To do so, a univariable analysis was performed in advance, and all variables with a p value <0.1 qualified for the multivariable model. Finally, C-statistics were performed to compare between the proposed classification and the infarction size regarding the predictive value for MACEs. All statistical analyses were performed using the SPSS software, version 22.0 (SPSS Inc., Chicago, Illinois, the U.S.A).

## Results

### Classification of the patients

A total of 190 patients were hospitalized during the study period. Among them, CMR data were unavailable in 10 patients (4 with atrial fibrillation, 1 with pacemaker implantation, 1 with end-stage renal failure, 2 with claustrophobia and 2 with poor-quality images.).

Of the enrolled 180 patients, 3 were classified as Grade 0 (1.7%), 79 as Grade 1 (43.9%), 45 as Grade 2 (25.%), 43 as Grade 3 (23.9%) and 8 as Grade 4 (4.4%). There were 2 patients exhibiting LV expansion but without LVEF reduction, who were assigned to Grade 2, expanding this group to 47 patients (26.1%). Baseline characteristics are listed in Table 1.

**Table 1:** Baseline characteristics of differently graded patients.

	Grade 0	Grade 1	Grade 2	Grade 3	Grade 4	p value
**Number of patients**	3 (1.7%)	79 (43.9%)	47 (26.1%)	43 (23.9%)	8 (4.4%)	-
**Age (y)**	53.4±18.1	58.9±8.9	56.4±8.9	57.2±8.5	63.9±5.2	0.086
**Men**	3(100%)	70 (88.6%)	43 (91.5%)	37(86.1%)	7 (87.5%)	0.423
**HTN**	1 (33.3%)	45 (56.7%)	19 (40.4%)	25 (58.1%)	7(87.5%)	0.142
**DM**	1 (33.3%)	26 (32.9%)	19 (40.4%)	18 (41.8%)	6 (75%)	0.095
**Hyperlipidemia**	2 (66.6%)	38 (48.1%)	20 (42.6%)	21 (48.8%)	5 (62.5%)	0.746
**Smoking**	0 (0%)	63 (79.7%)	35(74.4%)	32 (74.4%)	7 (87.5%)	0.093
**Renal insufficient**	0 (0%)	2 (2.5%)	1 (2.1%)	2 (4.6%)	1(12.5%)	0.864
**Culprit vessel**						
**LAD**	2(66.7%)	28 (35.4%)	24 (51.1%)	35 (81.4%)	5 (62.5%)	<0.001
**LCX**	0 (0%)	13 (16.5%)	8 (17%)	4 (9.3%)	2 (25%)	<0.001
**RCA**	1 (33.3%)	38 (48.1%)	15 (31.9%)	4 (9.3%)	1 (12.5%)	<0.001
**Multivessel disease**	1 (33.3%)	33 (41.8%)	16 (34.1%)	26 (60.5%)	6(75%)	0.03
**Reperfusion therapy**						
**PPCI**	2(66.7%)	41 (51.9%)	23(48.9%)	23 (53.5%)	5(62.5%)	0.863
**Pharmacoinvasive**	1 (33.3%)	38 (48.1%)	24 (51.1%)	20 (46.5%)	3 (37.5%)	0.431
**Reperfusion time (h)**	4.86±0.41	5.11±2.45	5.81±3.34	5.76±3.01	5.88±2.99	0.665
**CMR time (d)**	5.22±1.81	5.56±1.83	5.23±2.01	5.34±2.71	7.32±1.45	0.093
**Infarction size (%LV)**	0±0	15.68±8.48	23.68±9.32	36.12±11.35	40.66±14.33	<0.001
**Peak CPK (IU/L)**	232±33	2136±144	3447±236	5448±199	6529±338	<0.001
**LVEF (%)**	61±1.42	61.37±5.39	48.4±4.90	41±8.45	38.35±8.47	<0.001

Values are presented as number (%) and/or Mean ± SD. HTN: Hypertension; DM: Diabetes mellitus; LAD: left anterior descending coronary artery; LCX: left circumflex coronary artery; RCA: right coronary artery; CMR: cardiac magnetic resonance imaging; y: year; d: day. LV: left ventricle; CPK: Creatine Phosphate Kinase; LVEF: left ventricular ejection fraction.

### Prognosis at 90 days among differently graded patients

**Fig 3:**
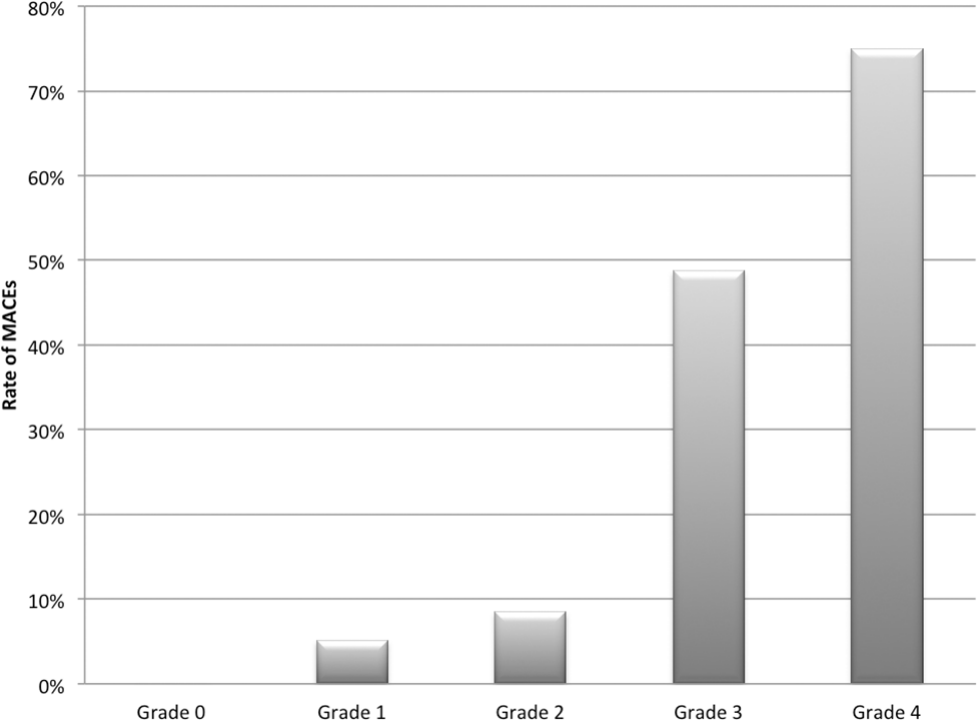
Rates of 90-day MACEs (defined as any death, resuscitated cardiac arrest, acute heart failure or stroke) were incremental with higher grades.

To demonstrate the general relationship between the new classification and prognosis, the rates of 90-day MACEs were compared across differently graded patients. As shown in Fig 3, during the follow-up, MACEs occurred incrementally from Grade 0 to Grade 4 (0 patient, 0%; 4 patients, 5.1%; 4 patients, 8.5%, 21 patients, 48.8% and 6 patients, 75%, respectively, p<0.001 by Fisher’s exact test). There were 4 deaths during the follow-up: one Grade-3 patient died of severe pulmonary edema during hospitalization; one Grade-3 and two Grade-4 patients suffered sudden death after discharge.

**Fig 4:**
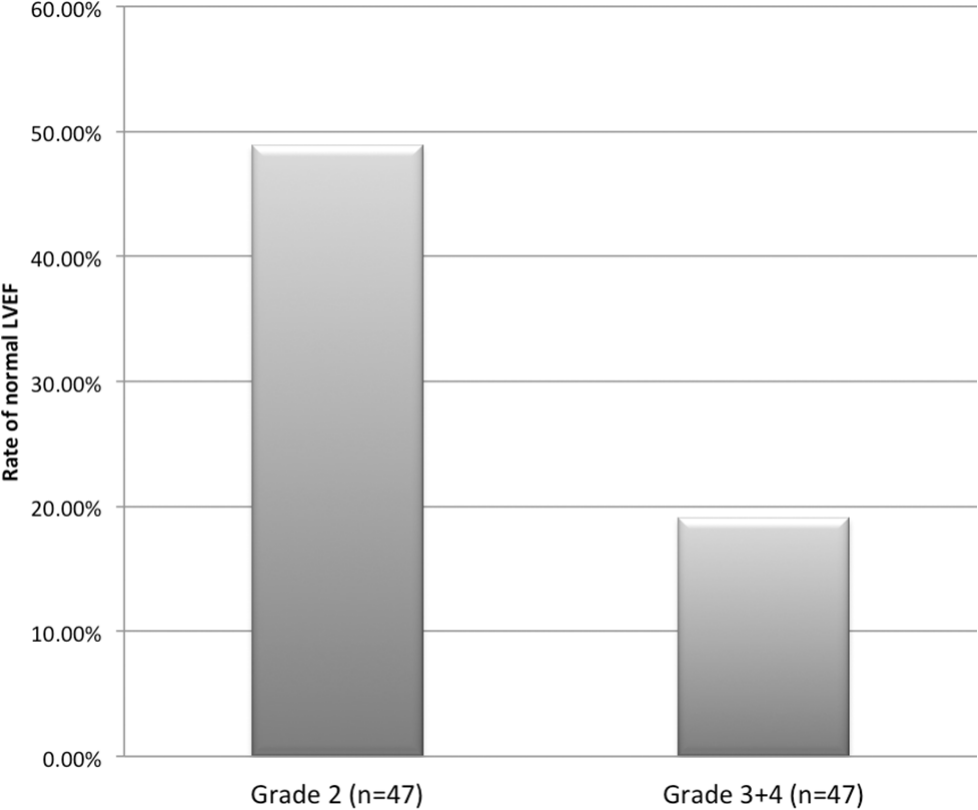
Echocardiographic follow-up at 90 days. Nearly half of the Grade-2 patients had LVEF recovered to normal (≥55%), while much fewer patients had improved LVEF in both Grade-3 and Grade-4 groups.

Subtracting the 4 deaths, all 176 patients underwent the second echocardiography after 90 days. Among the patients with reduced LVEF, 23 of the 47 (48.9%) Grade-2 patients had a recovered LVEF (≥55%). On the contrary, only 8 Grade-3 and 1 Grade-4 patient (19.1% of the 47 surviving patients in both groups) exhibited normal LVEF (p<0.001 compared with Grade-2 patients, by student’s t test, Fig 4).

### Relationship between the classification and infarction size

**Fig 5:**
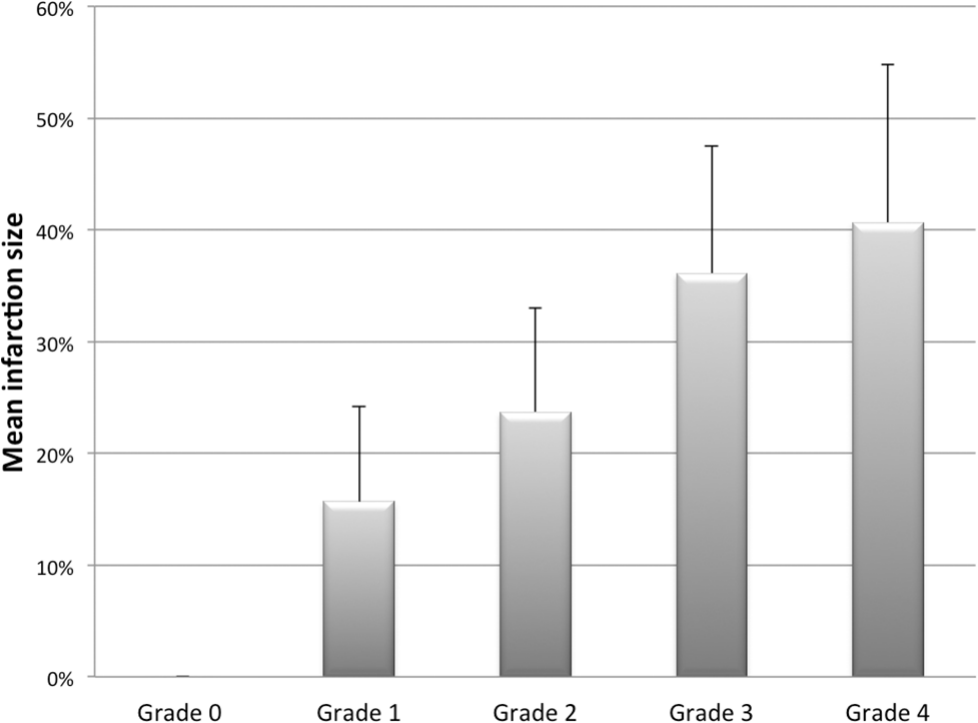
Infarction sizes (expressed as percentage of myocardial necrosis to left ventricular mass) increased remarkably in patients with higher grades.

Infarction extent has been proved to be the most potent prognostic factor of STEMI[17,18]. To clarify its correlation with the current classification, mean infarction sizes were compared among differently graded patients. As expected, highly-graded patients had significantly larger infarction sizes than those of lowly-graded patients (0±0%, 15.68±8.48%, 23.68±9.32%, 36.12±11.35% and 40.66±14.33% from Grade 0 to 4, respectively, p<0.001 by Chi-square test; Fig 5), demonstrating a positive relationship between the patient’s grade and the mean infarction size (coefficient = 0.623, p<0.001 by Spearman’s correlation test).

**Fig 6:**
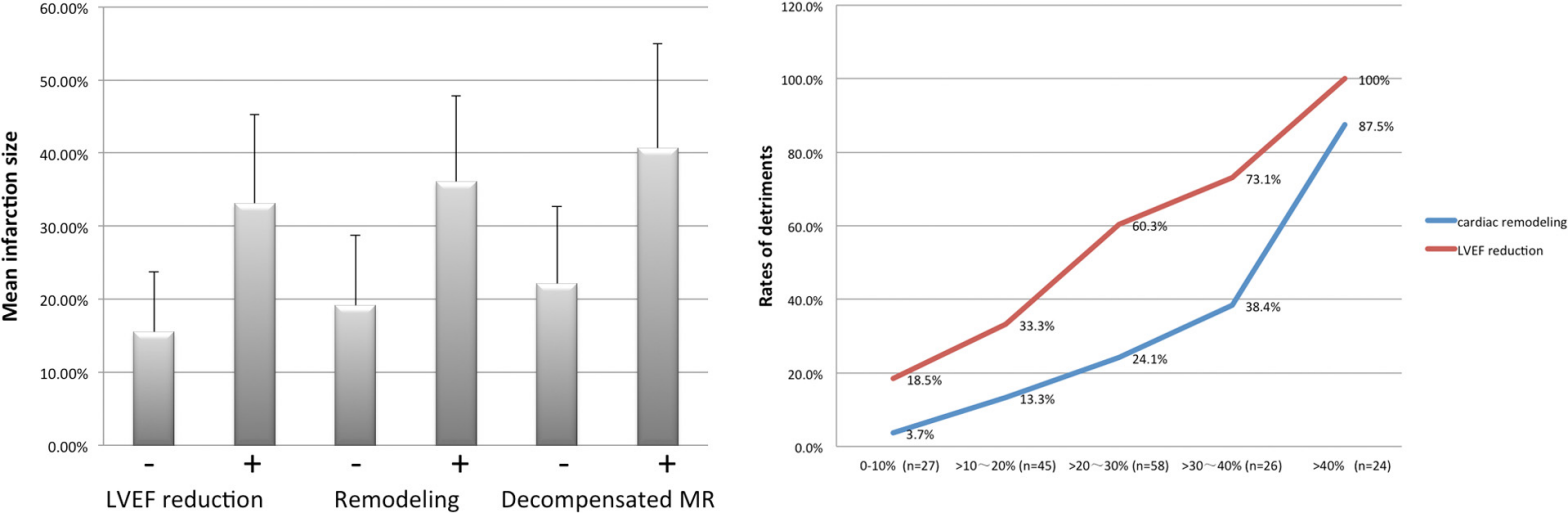
Infarction size is a key determinant of pathophysiological detriments. Left: Mean infarction sizes were compared between patients with or without LVEF reduction, cardiac remodeling and decompensated mitral regurgitation, respectively. Larger infarction size provoked the occurrence of all the detriments. LVEF: left ventricular ejection fraction, MR: mitral regurgitation. Right: Infarction sizes were stratified by every 10% increment of left ventricular myocardial volume. Larger infarction sizes were accompanied by significantly higher risks of both LVEF reduction and cardiac remodeling.

To explain the close relationship between the classification and the infarction size, the impacts of infarction size on individual classification criterion were analyzed. Notably, all functional and morphological detriments implicated by the classification (e.g. reduced LVEF, cardiac remodeling and decompensated mitral regurgitation) were remarkably evoked by larger infarction sizes (33.12±12.13% vs. 15.48±8.24% between the patients with and without reduced LVEF, p<0.001; 36.07±11.77% vs. 19.12±9.66% between the patients with and without cardiac remodeling, p<0.001; 40.66±14.33% vs. 22.11±10.54% between the patients with and without decompensated mitral regurgitation, p = 0.006; all by Student’s t-test, Fig 6 left). This was further verified by the fact that when the mean infarction sizes were stratified, the occurrence of LVEF reduction and cardiac remodeling increased dramatically from the lowest to the highest quartile (from 18.5% to 100% for LVEF reduction and 3.7% to 87.5% for cardiac remodeling, respectively, when infarction size was from 0–10% to >40%, both p<0.001 by Chi-square tests, Fig 6 right). Consistently, 7 of the 8 Grade-4 patients who exhibited decompensated mitral regurgitation had an infarction size >30%.

### Impact of mitral regurgitation on MACEs

Mitral regurgitation was observed from differently graded patients. To clarity differentiated prognostic influences of mitral regurgitation in different clinical setting, the rates of MACEs were compared among patients without mitral regurgitation, patients with apparent regurgitation but lacking cardiac remodeling (seen in Grade 0–2 patients), and patients with mitral regurgitation on the basis of severe morphological and functional deteriorations (Grade-4 patients). Mitral regurgitation did not manifest notable influence on MACEs when the normal shape of LV was persevered (0% compared with 15.7% in no-regurgitation patients, p = 0.136 by Student’s t test). On the opposite, mitral regurgitation concomitant with apparent cardiac remodeling was shown to correlate with significantly higher MACEs rates (75% compared with 15.7% in no-regurgitation patients, p = 0.005 by Fisher’s exact test).

### Prognostic value of the pathophysiological classification

**Fig 7:**
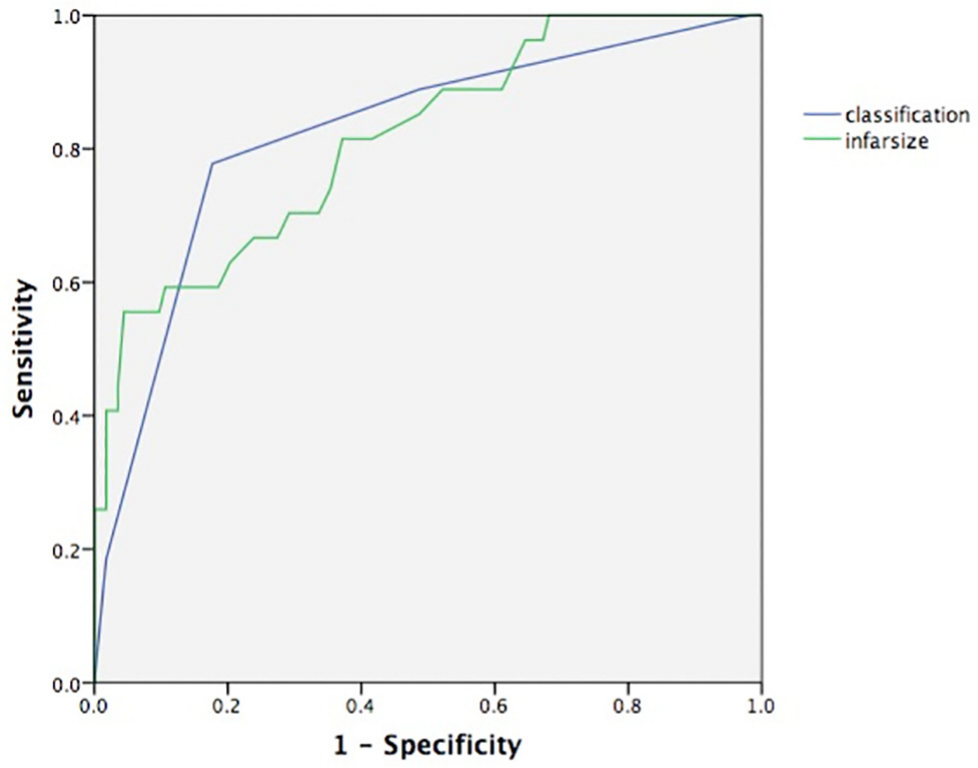
The pathophysiological classification demonstrates comparable prognostic values as infarction size by C-statistics in the prediction of 90-day MACEs.

To further validate the prognostic value of the pathophysiological classification, potential influential factors of prognosis were adjusted by logistic regression. Only the infarction size and patient’s grade remained to be independent predictors of 90-day MACEs (OR = 2.612 for the classification, p = 0.005; 1.083 for infarction size, p = 0.003, Table 2). Interestingly, C-statistics demonstrated a comparable predictive value between the two indicators (0.819 for the classification, 95% CI 0.763–0.875 vs. 0.813 for the infarction size, 95% CI 0.749 to 0.873, p = 0.876, Fig 7).

**Table 2:** Logistic regression analysis for the predictor of 90-day MACEs.

	Univariable analysis		Multivariable analysis	
Variables	OR	p value	OR	p value
**In-hospital classification**	2.056	<0.01	2.612	0.005
**Infarction size**	1.075	0.049	1.083	0.003
**Gender**	1.011	0.906	Not included	-
**Age**	1.052	0.246	Not included	-
**HTN**	1.956	0.244	Not included	-
**DM**	1.135	0.676	Not included	-
**Smoking**	3.977	0.191	Not included	-
**Hyperlipidemia**	1.401	0.504	Not included	-
**Culprit vessel**	1.205	0.342	Not included	-
**Reperfusion method**	1.211	0.770	Not included	-
**Multivessel disease**	1.806	0.303	Not included	-
**Reperfusion time**	1.089	0.336	Not included	-
**CMR time**	0.966	0.824	Not included	-
**LVEF**	0.957	0.233	Not included	

HTN: Hypertension; DM: Diabetes mellitus; LVEF: left ventricular ejection fraction

## Discussion

Recognition of patients who are seriously injured from STEMI is of very importance. Compared with those presenting less cardiac detriments, this population is remarkably subjective to adverse cardiovascular events, so that more frequent medical follow-up and more aggressive therapy is necessary for them. For example, the achievement of maximal titration of cardiac-protective medications is imperative. In this study, we demonstrated that the predictable pathophysiological changes might underlie a typical kind of criteria to objectively differentiate the severity of STEMI.

Patients will firstly be classified according to the detection of myocardial necrosis. This is essential because a very timely reperfusion or self-recanalization may leave patients without detectable necrosis [19,20]. Despite being at a slightly increased risk of MACEs in the acute phase due to augmented stiffness and reduced compliance of the left ventricle caused by myocardial edema [21–23], these so-defined Grade-0 patients compose a small group in the STEMI population–i.e. with the slightest cardiac injury.

Afterwards, the majority of STMEI patients who develop substantial myocardial necrosis can be further stratified by the concomitant functional and morphological abnormalities.

Comparing to the Grade-1 patients, who exhibit normal cardiac function and morphology, the reduction of LVEF is usually indicative of more extended injury, but the degree may vary in different scenario. Jeopardized pump function is more attributable to the stunned rather than necrotic myocardium in a relatively smaller infarction (i.e.Grade-2 patients). Nevertheless, extensive infarction often results in significant and quick morphological compensation, known as cardiac remodeling. In this regard, the worsening of LVEF seen in Grade-3 and Grade-4 patients is a reflect of both large necrosis and geometric deconstruction[24]. In the current study, cardiac remodeling was determined by increased LVEDV[25]. Using this definition, only two patients exhibited cardiac remodeling but preserved LVEF, who might have pre-STEMI ventricular enlargement due to other reasons. It is speculated that, with a proper cutoff point of the LVEDV, infarction-induced cardiac remodeling will have a tight concordance with LVEF reduction, thus guaranteeing a clear differentiation between the Grade-2 and the Grade-3 patients.

Finally, mitral regurgitation additional to functional and morphological ventricular deteriorations has been proved an explicit mark of cardiac decompensation [26,27] and therefore adapted as the criterion of the most severe cardiac detriments in the classification. Contrarily, mitral regurgitation based on a normal-shaped ventricle is not related to worse prognosis [28,29] and hence doesn’t indicate of more serious cardiac injury.

The value of the proposed classification is proved by its close relationship with the prognosis of current cohort. On one hand, the ascending grade is a strong indicator of higher rates of 90-day MACEs after adjusting other influential factors. This can be explained by the results of previous studies, that the presence of detectable myocardial necrosis[30], LVEF reduction[4,5], cardiac remodeling [31–34] and decompensated mitral regurgitation[26,27] will brings accumulating risks on patients. On the other hand, the classification also indicates of the probability of cardiac recovery. For instance, more Grade-2 patients have experienced LVEF improvement than both the Grade-3 and Grade-4 patients have at follow-up.

One important trait of the classification is its close relationship with infarction extent. This can be explained by our results that the occurrences of functional and morphological detriments are largely evoked by extended infarction. It is notable that, in terms of the prediction of 90-day MACEs, the classification has a comparable value as that of the infarction size itself, thus providing the classification an important advantage–namely, being a good surrogate of infarction extent but avoiding the requirements of complicated and facility-reliant quantification.

To be noted, the usage of CMR in this study was only to guarantee the equal criteria and comparison. In clinical practice, recognition of myocardial necrosis can be based on other methods according to the time point and facility availability, in the meanwhile, functional and morphological parameters can be acquired through multiple cardiac imaging technologies.

## Study Limitations

Due to the high cost of CMR, the sample size of the study was limited. Moreover, the criteria may be further refined to achieve a more precise stratification.

## Conclusion

Based on different pathophysiological stages, the severity of STEMI can be objectively stratified while patient’s prognosis can be well indicated.
